# The Incidence of Cancer

**Published:** 1922-08

**Authors:** 


### THE INCIDENCE OF CANCER

"The imagination of the race," says a popular writer, "has ever endowed cancer with a peculiar individuality of its own. Although it has vaguely personified in darkest ages other diseases like the Plague, the Pestilence and Maya (the small pox) these have rapidly faded away in even the earliest light of civilization, and have never approached in concreteness and definiteness the malevolent personality of Cancer."[1] And today there is being added to the horror of the disease and the mystery of its geneses the widespread reputation that cancer is heavily on the increase in all civilized countries.[2]

The statistics of the United States registration area show a continuous increase in the reported cancer deaths, as do similar statistics in other countries. On this fact has been based a widespread belief that cancer is a growing menace, which has become an integral and disquieting part of the layman's knowledge of cancer. That this increase in the number of reported deaths from cancer does not necessarily mean an increase in the actual amount of human cancer has been pointed out frequently enough, but qualifying statements notoriously make less impression than unqualified conclusions. Five years ago, Willcox[3] analyzed the statistical data on which has been based the belief that cancer is increasing, and concluded that the evidence did not establish this conclusion. He called attention to the fact that a large part of the alleged increase was produced by the inclusion of more cases because of more accurate diagnosis; for when the statistics were so kept as to permit of analysis it was found that the increase was entirely in cancer in inaccessible parts of the body, whereas in the same population there is no increase in the number of cancers superficially located, so that their diagnosis could be made as accurately years ago as now. The increasing average age of the population in any community of necessity carries with it an increased cancer rate, as also does more accuracy in reporting deaths, especially the elimination of old age as a cause of death. Hence we find the cancer

---

[1] Hutchinson, W.: Preventable Diseases, Boston, Houghton, Mifflin Company, 1914, p. 350.

[2] Fisher, Irving, and Fisk, E. L.: How to Live, New York, 1919, p. 381.

[3] Willcox, W. F.: On the Alleged Increase of Cancer, J. Cancer Res. **2**: 267 (July) 1917.

death rate highest in the most advanced communities, and lowest in the most backward, not because "cancer is a disease of civilization," but simply because in the former localities the statistics come nearer reflecting the actual facts. As an illustration of the influence of improved diagnosis on the mortality statistics may be cited the fact that, between 1900 and 1915, the reported appendicitis mortality showed an increase of 40 per cent., while at the same time the real rate was undoubtedly decreasing because of improved diagnosis and treatment. Increasing accuracy in diagnosis works only in one way; that is, it results in attributing to cancer many deaths which would formerly have been incorrectly attributed to some other cause, and changes but few in the other direction.

Insurance statistics are perhaps the most valuable we have in this country, for there is likely to be more care taken in giving the true cause of death when payment of insurance claims is involved. When, therefore, a competent statistician accustomed to study figures of facts without regard to tradition and unmoved by superficial guesswork applies rigid logic to premises which have been shorn of some of their obvious errors, he deserves a respectful hearing, even when "common knowledge" appears to be against him. Strong[4] has recently concluded that we can not now determine whether the cancer mortality is slightly increasing, practically stationary, or slightly decreasing, but that we can be sure it is not greatly increasing. A more exact result is something for future investigations when reliable statistics for a long period of years are obtainable. Fair-minded students of mortality statistics have often pointed out that with the development of medical science there has ensued a gradual increase in the correctness of diagnosis, of the cause of death as well as a more complete record-

---

[4] Strong, W. M.: Is Cancer Mortality Increasing? J. Cancer Res. **6**: 251 (July) 1921.

ing of all cases of disease. The imperfections of the records of even a generation ago need not be dwelt on here. Strong reminds us that undoubtedly in the past many deaths attributed to old age should properly have been set down as cancer, and many others attributed to other causes would undoubtedly have been attributed to cancer if the correct diagnosis had been made. The result of this would be that if there were really stationary cancer mortality it would nevertheless appear to be increasing considerably because of the increasing correctness of diagnosis.

Strong has requisitioned the data from two of the largest life insurance companies secured during the years 1911 to 1921, inclusive, which may be regarded as relatively accurate in comparison with figures from other sources. The comparative novelty in his interpretation of available cancer mortality statistics lies in the insistence on comparing death rates for the same ages, as cancer is an older age disease. Influx or emigration of young persons would unquestionably alter the conclusions from time to time if an entire population were made the basis of deductions, as is usually done.

In view of all the tendencies that exist to cause an appearance of statistical increase of cancer mortality, the failure of careful statistical study to indicate any noteworthy increase of cancer is strong evidence that such an increase is not taking place. This conclusion, of course, does not lessen the seriousness of the cancer problem; but it should prevent fears, at present not justified, that it is an increasing menace that threatens to overwhelm the human race, as some alarmists have maintained. There will always be plenty of other means of death besides cancer.

In combating the fear of a growing menace of cancer, it has been remarked [1] that a disease for which 80 per cent. of the mortality occurs after 45 years of age is scarcely likely to threaten the continued existence of the race. Let us frankly realize, however, that meanwhile the statistics of the United States registration area show a continuous increase in cancer deaths. The hopeful confidence of a seriously minded profession supporting a host of tremendously earnest investigators in the field of cancer research may be regaled by the assurance that cancer is not gaining a greater foothold in the younger members of our population; yet the problem of the unphysiologic deaths of even the later years of life continues to loom so large and difficult that the unceasing, unrelenting, undismayed attack on it through every resource of science calls for great professional courage as well as the highest intelligence that man can mobilize.—*Journal A. M. A.*, June 10, 1922.

